# Effects of a Brief Inpatient Mindfulness‐Based Stress Reduction Program on Psychological Distress and Marital Satisfaction in High‐Risk Pregnancy: A Randomized Controlled Trial

**DOI:** 10.1002/hsr2.73154

**Published:** 2026-09-23

**Authors:** Solmaz Heydarifard, Faezeh Safaralinezhad, Atefeh Safaralinezhad

**Affiliations:** ^1^ Social Determinants of Health Research Center, Research, Institute for Prevention of Non‐Communicable Diseases Qazvin University of Medical Sciences Qazvin Iran

**Keywords:** high‐risk pregnancy, marital satisfaction, mindfulness‐based stress reduction, perinatal mental health, randomized controlled trial

## Abstract

**Objective:**

Hospitalized women with high‐risk pregnancies are particularly vulnerable to psychological distress and relational strain. This randomized controlled trial aimed to evaluate the effectiveness of a brief inpatient mindfulness‐based stress reduction (MBSR) program in improving psychological distress and marital satisfaction.

**Methods:**

Fifty‐seven pregnant women (24–28 weeks' gestation) hospitalized for premature rupture of membranes were randomized to either an 8‐week brief MBSR program or treatment‐as‐usual. Fifty‐three participants completed the study. Outcomes included psychological distress assessed by the distress subscale of the Mental Health Inventory (MHI‐28), mindfulness measured by the Five Facet Mindfulness Questionnaire (FFMQ), and marital satisfaction assessed by the Kansas Marital Satisfaction Scale (KMSS), assessed at baseline, post‐intervention, and 4‐week postpartum follow‐up. Repeated‐measures ANOVA with Greenhouse–Geisser correction was performed.

**Results:**

Significant time × group interactions were observed for mindfulness, anxiety, depression, and marital satisfaction (all *p* < 0.001). Participants in the intervention group showed sustained improvements in mindfulness and marital satisfaction and significant reductions in anxiety and depressive symptoms compared with controls.

**Conclusion:**

A brief inpatient MBSR program may reduce psychological distress and improve marital satisfaction among hospitalized women with high‐risk pregnancies. Larger multicenter trials with extended follow‐up are warranted.

## 1 Introduction

Pregnancy represents a critical developmental period characterized by heightened biological, psychological, and social transitions that shape both maternal well‐being and infant outcomes [1]. During pregnancy, women may experience challenges related to changing personal roles, interpersonal relationships, and psychological adjustment [2]. Pregnancy can be particularly stressful, especially for first‐time mothers, and may increase the risk of psychological problems such as anxiety and depressive symptoms [3].

Women experiencing high‐risk pregnancies often require prolonged hospitalization. This situation may expose them to additional psychological challenges, including anxiety, isolation, uncertainty, and disruption of daily routines, which can negatively affect maternal mental health [4, 5] Such psychological strain may negatively affect marital satisfaction, leading to communication difficulties and increased relational conflict [6, 7]. Conversely, marital satisfaction and partner support during pregnancy have been associated with maternal psychological well‐being and family‐related outcomes [8, 9, 10]. According to Mercer's theory of motherhood, experiences of commitment, attachment, and readiness enhance maternal–fetal bonding, which also impacts marital satisfaction [11].

Mindfulness‐based programs have emerged as promising interventions to support maternal mental health and strengthen marital relationships. These practices enhance emotional regulation, empathy, and stress management while fostering positive connections between parents and their unborn child [12]. Mindfulness may also enhance interpersonal awareness and communication within couples to recognize perspectives and challenges within their marriage, promoting constructive communication and improving relational quality [13, 14]. It involves sustained attention to the present moment, allowing individuals to respond reflectively rather than automatically [15]. Structured programs, such as Mindfulness‐Based Stress Reduction (MBSR), provide participants with guided meditation, body scanning, and gentle physical exercises to cultivate awareness of thoughts, feelings, and bodily sensations [16, 17].

Despite growing evidence supporting mindfulness‐based interventions during pregnancy, several critical gaps remain. First, most existing studies have focused on community‐based or low‐risk pregnant populations, with limited attention to hospitalized women experiencing high‐risk obstetric conditions such as premature rupture of membranes. These women are exposed to prolonged medical uncertainty, restricted mobility, and elevated psychological vulnerability, potentially amplifying both individual distress and relational strain. Second, while reductions in anxiety and depression have been widely examined, fewer studies have investigated relational outcomes—particularly marital satisfaction—as a clinically meaningful dimension of perinatal well‐being. Given the well‐documented bidirectional relationship between maternal mental health and couple functioning, examining both psychological and relational outcomes within a single randomized controlled framework is essential. Finally, the feasibility and potential impact of brief, structured mindfulness sessions integrated into inpatient obstetric care settings remain underexplored. Addressing these gaps may contribute to a more comprehensive and clinically applicable model of perinatal psychosocial care.

Given the potential impact of pregnancy‐related stress on maternal mental health and marital satisfaction—and the underutilization of preventive psychosocial interventions—this study aimed to evaluate whether brief structured mindfulness training could enhance mental well‐being and marital satisfaction in hospitalized expectant mothers. This study may provide evidence for the implementation of mindfulness‐based programs as a supportive strategy in clinical settings for pregnant women. To our knowledge, no prior randomized controlled trial has simultaneously examined psychological and marital outcomes in a hospitalized high‐risk pregnancy population using a structured inpatient mindfulness protocol. We hypothesized that participants receiving the MBSR program would show increased mindfulness and marital satisfaction and reduced anxiety and depression compared to controls.

## 2 Materials and Methods

### 2.1 Study Design

**Figure 1:**
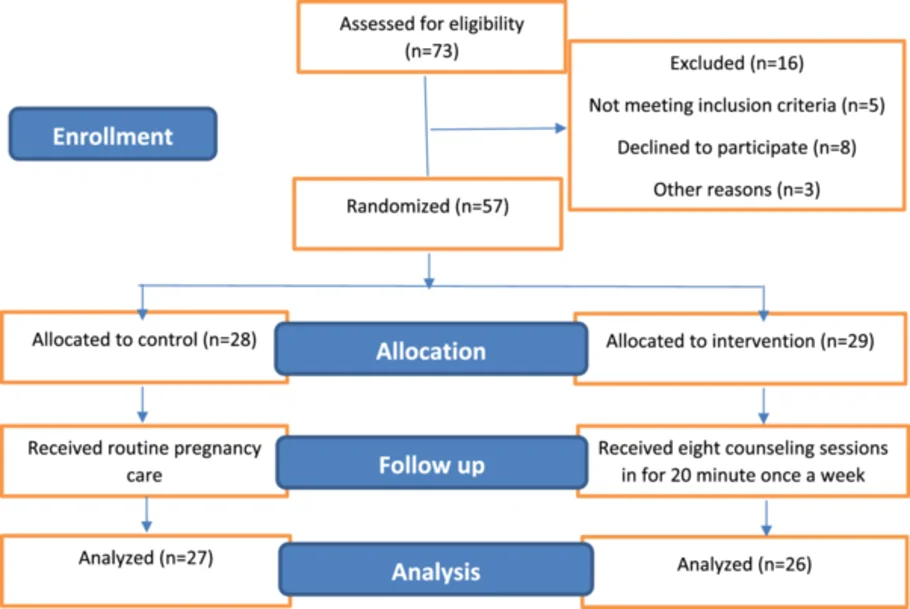
CONSORT flow diagram of patients included in the study.

This randomized controlled trial (RCT) was registered in the Iranian Registry of Clinical Trials (IRCT20241213064043N1). This hospital‐based randomized controlled trial (RCT) compared an 8‐week Mindfulness‐Based Stress Reduction (MBSR) intervention with a treatment‐as‐usual (TAU) control group. The study adhered to the ethical principles of the Declaration of Helsinki (1964) and its subsequent amendments, and all participants provided written informed consent prior to enrollment. The study was reported in accordance with the CONSORT guidelines (Figure 1). This study was approved by the Institutional Ethics Committee (Approval No: IR. QUMS. REC.1403.355).

### 2.2 Participants

Eligible participants were women meeting the predefined inclusion criteria. Inclusion criteria included the following: (1) Pregnant women 24 to 28 weeks with premature rupture of membranes admitted to the hospital, (2) Willingness to participate in the study, (3) Iranian and able to speak and understand Persian, (4) Possessing basic literacy skills. Basic literacy skills were assessed during the initial screening by asking participants about their ability to read and write Persian. Furthermore, participants' ability to independently complete the study questionnaires confirmed their basic literacy skills. Exclusion criteria included: (1) diagnosed mental disorders under medication, (2) concurrent participation in other counseling sessions, (3) recent life‐threatening events (e.g., accident or family loss within 3 months), (4) unwillingness to participate, or (5) pregnancy termination before completing at least 60% of the sessions. Detailed baseline demographic and obstetric characteristics of the participants are presented in Supporting Information Table S1. No statistically significant differences were observed between the intervention and control groups for any baseline characteristic.

### 2.3 Sample Size and Power Analysis

An a priori power analysis was conducted using G*Power version 3.1.9.2 to determine the minimum required sample size for detecting a clinically meaningful group × time interaction effect in a repeated‐measures design. Given the limited number of randomized controlled trials focusing specifically on hospitalized women with high‐risk pregnancies, the effect size estimation was based on previous mindfulness‐based intervention studies in perinatal populations reporting moderate‐to‐large effects on psychological outcomes. The sample size calculation indicated that a minimum of 56 participants was required based on an effect size (Cohen's d = 0.88) reported by Tavousi et al. (2024), with an alpha level of 0.05 and statistical power of 80%. To account for potential attrition, additional participants were recruited beyond the calculated sample size. A total of 57 participants were randomized, and 53 participants completed the study and were included in the final analysis.

Due to the restricted pool of eligible hospitalized women with premature rupture of membranes during the recruitment period, consecutive sampling was used until the calculated sample size was achieved. A total of 57 participants were ultimately enrolled and randomized. Four participants were lost to follow‐up; however, the final sample size remained close to the target calculated in the a priori power analysis. Random allocation was performed using a computer‐generated randomization sequence with concealed allocation. To minimize contamination between groups, intervention sessions were provided only to participants allocated to the intervention group, and control participants did not have access to the MBSR sessions or intervention materials during the study period. Due to the nature of the intervention, participants and intervention providers were not blinded; however, data analysis was conducted by a researcher blinded to group allocation.

### 2.4 Intervention

The intervention group attended 8 weekly, in‐person mindfulness‐based counseling sessions (20–25 min each) based on the standardized MBSR protocol by Kabat‐Zinn (1990) [15, 18]. Each session followed a structured MBSR‐based protocol adapted for hospitalized women with high‐risk pregnancies. The sessions included psychoeducation about stress and mindfulness, attention to present‐moment experiences, mindful breathing exercises, body awareness practices, and techniques aimed at improving emotional regulation and coping with pregnancy‐related stress. The content was delivered progressively across sessions, beginning with basic mindfulness concepts and advancing toward the application of mindfulness skills in managing distressing thoughts and emotions. Each session included guided meditation, body scanning, gentle physical exercises, and reflection on thoughts and emotions. Participants received individualized feedback from the researcher on assigned exercises. All sessions were delivered by the same trained researcher to ensure consistency in intervention delivery. The control group received standard care without counseling. The MBSR sessions are summarized in Table 1. The control group did not receive any counseling training during the study. The intervention duration and format were selected to ensure feasibility within the inpatient setting, where women with high‐risk pregnancies may have limited physical capacity and emotional resources for lengthy sessions. The choice of an inpatient, brief‐format intervention was based on the clinical characteristics of the target population, as hospitalized women with high‐risk pregnancies may experience elevated psychological distress while having limited opportunity to participate in lengthy outpatient programs.

**Table 1:** Mindfulness intervention sessions.

Session	Content summary
1	Basics and principles of mindfulness; introduction to meditation focusing on breath and external awareness
2	Mindfulness‐promoting concepts; “wise mind”; calming mindful movements; seated meditation; breath awareness.
3	Distinction between mindfulness and its absence; meditation on external awareness and wise mind; mood exercise exploring thoughts from multiple perspectives.
4	Mindful listening; four‐dimensional meditation; self‐care strategies; 3‐min breathing break; overcoming meditation challenges.
5	Meditation on external awareness and wise mind.
6	Muscle relaxation; clearing the mind; conscious gratitude exercises.
7	Review of previous exercises; practice mindfulness methods for immediate stress reduction; four‐dimensional meditation.
8	Consolidation of meditation techniques; stress alleviation exercises; four‐dimensional meditation.

### 2.5 Procedures

Participants completed a demographic and obstetric characteristics questionnaire, the Mental Health Inventory (MHI‐28) [19], the Kansas Marital Satisfaction Scale (KMSS) [20, 21], and the Five‐Factor Mindfulness Questionnaire (FFMQ) [22, 23] at three time points: T0 (Baseline), T1 (post‐intervention), and T2 (4 weeks postpartum). The 4‐week postpartum assessment was selected to evaluate whether the effects of the intervention were maintained beyond pregnancy and during the early postpartum transition.

## 3 Outcomes/Measures

### 3.1 Demographic and Obstetric Characteristics

Participants completed a survey collecting demographic and obstetric characteristics, including age, education level, number of children and pregnancies, gestational age, employment status, economic status, marital status, length of hospitalization, and reason for hospitalization.

### 3.2 Primary Outcome

The primary outcome was psychological distress, assessed using the Mental Health Inventory (MHI‐28). The MHI‐28 consists of two dimensions: psychological distress (items 15–28), which reflects anxiety and depressive symptoms, and psychological well‐being (items 1–14). In this study, the psychological distress subscale was considered the primary psychological outcome, while the psychological well‐being subscale was analyzed as a complementary indicator of mental health status [18]. This 28‐item questionnaire includes two subscales: psychological well‐being (items 1–14) and psychological distress (items 15–28). Responses are rated on a five‐point Likert scale (1 = never, 5 = always), with subscale scores ranging from 14 to 70. The Persian version of MHI‐28 demonstrates good internal consistency (Cronbach's alpha = 0.89) and validated two‐factor structure [19]. The psychological distress and psychological well‐being subscales were analyzed separately to provide a comprehensive assessment of participants' mental health status.

### 3.3 Secondary Outcome

The secondary outcome was marital satisfaction, assessed using the Kansas Marital Satisfaction Scale (KMSS) [20]. The KMSS is a brief three‐item questionnaire scored on a seven‐point Likert scale (1 = very dissatisfied, 7 = very satisfied), with higher scores indicating greater marital satisfaction. A total score of 17 or above reflects marital satisfaction. The Persian version has demonstrated excellent reliability (Cronbach's alpha = 0.98) and confirmed construct validity [21].

### 3.4 Exploratory Outcome

Mindfulness was evaluated as an exploratory outcome using the Five‐Factor Mindfulness Questionnaire (FFMQ) [22, 23]. The FFMQ consists of 39 items measuring five facets: observing, describing, acting with awareness, non‐judgment of internal experiences, and non‐reactivity to internal experiences. Responses are rated on a five‐point scale (1 = never, 5 = always). The Persian adaptation demonstrated strong test‐retest reliability (0.76–0.86 across subscales, 0.89 overall) and adequate construct validity (RMSEA = 0.07, accounting for 49% of variance) [23].

### 3.5 Statistical Analysis

All statistical analyzes were performed using SPSS version 26. Analyzes were conducted on participants who completed the study (per‐protocol analysis). Descriptive statistics included means and standard deviations for continuous variables and frequencies (%) for categorical variables. The normality of continuous data was assessed using the Kolmogorov‐Smirnov test. Effect sizes were interpreted according to conventional benchmarks (0.01 small, 0.06 medium, 0.14 large).

Baseline differences between groups were evaluated using the independent‐samples *t*‐test for normally distributed continuous variables and the chi‐square test for categorical variables. For non‐normally distributed data, the Mann‐Whitney *U* test and Fisher's exact test were employed.

To examine the effects of the intervention on outcomes over time (T0: baseline, T1: post‐intervention, T2: 4 weeks postpartum), repeated‐measures analysis of variance (ANOVA) was conducted. Results were reported with a 95% confidence interval, and statistical significance was set at *p* < 0.05.

## 4 Results

### 4.1 Participant Characteristics and Baseline Comparability

A total of 57 participants were randomized, of whom 53 completed the study and were included in the final analysis (intervention: *n* = 27; control: *n* = 26). The mean age of participants in the control group was 29.85 years (SD = 3.78), while the mean age in the intervention group was 28.59 years (SD = 5.04).

Baseline demographic and obstetric characteristics, including education level, gravidity, history of miscarriage, employment status of participants and their spouses, economic status, duration of hospitalization, cause of hospitalization, and pregnancy intention, were comparable between the intervention and control groups. No statistically significant between‐group differences were observed for any baseline variables, indicating that randomization successfully produced equivalent groups prior to the intervention (Supporting Information Table S1).

### 4.2 Descriptive Statistics of Study Variables

Table 2 presents the means and standard deviations of psychological distress (including anxiety and depressive symptoms), mindfulness, and marital satisfaction scores for the intervention and control groups at pre‐test, post‐intervention, and 4 weeks postpartum.

**Table 2:** Descriptive statistics of study variables at pre‐test, post‐intervention, and four weeks postpartum.

Variable	Time point	Control mean (SD)	Intervention mean (SD)
Mindfulness	Pre‐test	112.75 (2.64)	113.21 (2.31)
Mindfulness	Post‐intervention	117.07 (2.90)	126.43 (2.89)
Mindfulness	Four weeks postpartum	116.21 (2.83)	125.36 (2.16)
Anxiety	Pre‐test	20.42 (4.01)	19.07 (3.65)
Anxiety	Post‐intervention	19.01 (3.71)	15.04 (2.48)
Anxiety	Four weeks postpartum	22.28 (4.35)	15.79 (2.27)
Depression	Pre‐test	21.32 (3.71)	23.09 (3.59)
Depression	Post‐intervention	20.23 (3.15)	14.87 (2.05)
Depression	Four weeks postpartum	24.30 (3.73)	15.76 (2.16)
Marital satisfaction	Pre‐test	66.45 (5.44)	64.18 (5.92)
Marital satisfaction	Post‐intervention	65.11 (6.62)	75.05 (5.19)
Marital satisfaction	Four weeks postpartum	63.23 (5.61)	69.99 (6.98)

At baseline, the two groups demonstrated comparable scores across all study variables. Following the intervention, participants in the MBSR group showed notable improvements in mindfulness and marital satisfaction, alongside significant reductions in anxiety and depression scores. These changes were maintained at the 4 weeks postpartum assessment. In contrast, the control group exhibited minimal changes across measurement points.

### 4.3 Assumption Testing

#### 4.3.1 Normality

Normality of the data was evaluated using skewness and kurtosis indices (Table 3). According to the criteria proposed by George and Mallery (2021) [24], distributions with skewness and kurtosis values within ±2 are considered approximately normal. All values for mindfulness, anxiety, depression, and marital satisfaction across groups and time points fell within this acceptable range, indicating that the assumption of normality was satisfied.

**Table 3:** Skewness and kurtosis indices for study variables.

Variable	Time point	Control skewness	Control kurtosis	Intervention skewness	Intervention kurtosis
Mindfulness	Pre‐test	0.271	0.368	−0.126	−1.361
Mindfulness	Post‐intervention	−1.532	1.689	−0.145	−1.550
Mindfulness	Four weeks postpartum	−1.042	−0.032	0.021	−0.779
Anxiety	Pre‐test	−0.021	−0.542	−0.077	−0.690
Anxiety	Post‐intervention	0.368	−0.169	0.501	−0.572
Anxiety	Four weeks postpartum	−0.112	−0.193	0.052	−0.449
Depression	Pre‐test	−0.206	−1.029	−0.188	0.292
Depression	Post‐intervention	1.476	1.581	0.253	−0.795
Depression	Four weeks postpartum	0.004	1.086	−0.115	−1.007
Marital satisfaction	Pre‐test	0.379	0.957	−0.153	−0.540
Marital satisfaction	Post‐intervention	0.699	1.186	0.289	−0.013
Marital satisfaction	Four weeks postpartum	0.379	−0.037	−1.846	1.374

#### 4.3.2 Homogeneity of Variance–Covariance Matrices

The Box's M test was conducted to examine the homogeneity of variance–covariance matrices. As shown in Table 4, the Box's M statistic was not statistically significant (Box's M = 17.78, F = 1.74, *p* = 0.064), supporting the assumption of equal variance–covariance matrices across groups.

**Table 4:** Box's M test for homogeneity of variance–covariance matrices.

Box's M	F	df	*p* value
17.783	1.736	78	0.064

#### 4.3.3 Homogeneity of Error Variances

Levene's test results indicated that the assumption of homogeneity of error variances was met for all study variables at pre‐test, post‐intervention, and 4 weeks postpartum (all *p* values > 0.05; Table 5).

**Table 5:** Levene's test for homogeneity of error variances.

Variable (Time point)	F	df1	df2	*p* value
Mindfulness (Pre)	1.306	1	54	0.258
Mindfulness (Post)	1.325	1	54	0.255
Mindfulness (Four weeks postpartum)	1.266	1	54	0.265
Anxiety (Pre)	0.161	1	54	0.690
Anxiety (Post)	0.074	1	54	0.786
Anxiety (Four weeks postpartum)	0.177	1	54	0.676
Depression (Pre)	0.622	1	54	0.434
Depression (Post)	0.631	1	54	0.431
Depression (Four weeks postpartum)	0.600	1	54	0.442
Marital satisfaction (Pre)	1.407	1	54	0.241
Marital satisfaction (Post)	0.929	1	54	0.340
Marital satisfaction (Four weeks postpartum)	1.370	1	54	0.247

#### 4.3.4 Sphericity

Mauchly's test of sphericity revealed significant violations of the sphericity assumption for all variables (*p* < 0.05; Table 6). Accordingly, Greenhouse–Geisser corrections were applied to all repeated‐measures analyzes.

**Table 6:** Mauchly's test of sphericity.

Variable	W	Chi‐square	df	*p* value
Mindfulness	0.852	8.516	2	0.014
Anxiety	0.803	11.660	2	0.003
Depression	0.799	11.891	2	0.003
Marital satisfaction	0.848	8.748	2	0.013

#### 4.3.5 Effects of the Intervention

Repeated‐measures analysis of variance (RM‐ANOVA) with Greenhouse–Geisser correction was conducted to evaluate the effects of time, group, and time × group interaction on mindfulness, anxiety, depression, and marital satisfaction (Table 7).

**Table 7:** Repeated‐measures ANOVA results (Greenhouse–Geisser corrected).

Variable	Source	Sum of squares	Mean square	F	*p* value	Partial η^2^
Mindfulness	Time	2589.083	1486.692	332.750	< 0.001	0.860
Mindfulness	Time × Group	720.750	413.866	92.631	< 0.001	0.632
Anxiety	Time	222.791	133.394	16.062	< 0.001	0.229
Anxiety	Time × Group	184.466	110.447	13.229	< 0.001	0.198
Depression	Time	607.563	364.832	35.315	< 0.001	0.395
Depression	Time × Group	782.131	469.657	45.462	< 0.001	0.457
Marital satisfaction	Time	679.019	391.165	10.779	< 0.001	0.166
Marital satisfaction	Time × Group	1123.922	647.461	17.841	< 0.001	0.248

#### 4.3.6 Mindfulness

A significant main effect of time was observed for mindfulness (F = 332.75, *p* < 0.001, partial η^2^ = 0.86), as well as a significant time × group interaction (F = 92.63, *p* < 0.001, partial η^2^ = 0.63), indicating that changes in mindfulness scores over time differed significantly between the intervention and control groups.

#### 4.3.7 Anxiety

For anxiety, a significant main effect of time was observed (F = 16.06, *p* < 0.001, η^2^ = 0.23), along with a significant time × group interaction (F = 13.23, *p* < 0.001, η^2^ = 0.20), demonstrating differential anxiety trajectories between groups across the three assessment points.

#### 4.3.8 Depression

Results for depression indicated a significant main effect of time (F = 35.32, *p* < 0.001, η^2^ = 0.40) and a significant time × group interaction (F = 45.46, *p* < 0.001, η^2^ = 0.46), suggesting that the intervention produced distinct changes in depressive symptoms relative to the control condition.

#### 4.3.9 Marital Satisfaction

A significant main effect of time was also found for marital satisfaction (F = 10.78, *p* < 0.001, η^2^ = 0.17), as well as a significant time × group interaction (F = 17.84, *p* < 0.001, η^2^ = 0.25), indicating that marital satisfaction evolved differently across time in the two groups.

#### 4.3.10 Post‐Hoc Comparisons

Bonferroni‐adjusted post‐hoc analyzes were conducted to examine between‐group differences (Table 8). Significant differences were observed between the intervention and control groups for all outcome variables.

**Table 8:** Bonferroni post‐hoc comparisons between groups.

Variable	Comparison	Mean Gifference	SE	*p* value
Mindfulness	Control—Intervention	−6.321	0.385	< 0.001
Anxiety	Control—Intervention	3.939	0.738	< 0.001
Depression	Control—Intervention	4.042	0.546	< 0.001
Marital Satisfaction	Control—Intervention	−4.808	1.034	< 0.001

Compared with the control group, participants in the MBSR group demonstrated significantly higher mindfulness scores (mean difference = −6.32, *p* < 0.001) and marital satisfaction scores (mean difference = −4.81, *p* < 0.001), as well as significantly lower anxiety (mean difference = 3.94, *p* < 0.001) and depression scores (mean difference = 4.04, *p* < 0.001).

#### 4.3.11 Summary of Findings

Overall, the results indicate that the mindfulness‐based intervention produced statistically significant and sustained improvements in mindfulness and marital satisfaction, along with significant reductions in anxiety and depression among hospitalized pregnant women. These improvements were evident at post‐intervention and were maintained at 4 weeks postpartum, whereas no comparable pattern of change was observed in the control group.

## 5 Discussion

This randomized controlled trial examined the impact of a brief inpatient Mindfulness‐Based Stress Reduction (MBSR) program on mindfulness, psychological distress, and marital satisfaction among hospitalized women with high‐risk pregnancies. The findings indicate that participants who received the intervention demonstrated significant and sustained improvements in mindfulness and marital satisfaction, alongside reductions in psychological distress, reflected by decreased anxiety and depressive symptoms, compared with those receiving treatment‐as‐usual. Importantly, these changes were maintained at 4‐week postpartum follow‐up, suggesting that even relatively brief, structured mindfulness training may confer short‐term durability of benefit in vulnerable perinatal populations.

The observed increase in mindfulness among participants in the intervention group is consistent with prior research demonstrating that structured mindfulness‐based programs effectively enhance present‐moment awareness and non‐judgmental attention during pregnancy and other periods of heightened stress [18, 25, 26]. In medically complicated pregnancies requiring hospitalization, women are often confronted with uncertainty, restricted mobility, and perceived loss of control. Under such circumstances, cultivating attentional stability and emotional awareness may serve as adaptive coping mechanisms. From a theoretical perspective, mindfulness may influence psychological distress through cognitive and emotional regulation pathways. According to mindfulness‐based theoretical models, increased awareness of internal experiences without judgment can reduce automatic reactions to stressful thoughts and emotions, thereby decreasing maladaptive coping patterns such as rumination and emotional avoidance [15, 16]. Evidence suggests that improvements in specific mindfulness facets—such as acting with awareness and non‐reactivity—are associated with reductions in psychological distress following mindfulness‐based interventions [27], which may help explain the concurrent symptom improvements observed in this study.

Consistent with this interpretation, the intervention group exhibited significant reductions in psychological distress over time, including improvements in anxiety and depressive symptoms assessed by the MHI‐28, whereas distress levels remained relatively stable or worsened in the control group. These findings align with prior evidence indicating that mindfulness‐based interventions reduce psychological distress, including anxiety and depressive symptoms, through enhanced emotion regulation, decreased rumination, and greater acceptance of internal experiences [18, 25, 26, 28]. These mechanisms are particularly relevant in high‐risk pregnancy, where uncertainty regarding maternal and fetal outcomes may increase threat perception and emotional reactivity. By promoting a more balanced response to stressors, mindfulness may help women reinterpret pregnancy‐related challenges and develop more adaptive emotional responses. Longitudinal research has also demonstrated that prenatal stressors and psychosocial conditions predict postpartum mental health outcomes [3], underscoring the importance of early preventive strategies during pregnancy. By strengthening adaptive emotional responses during a period of elevated vulnerability, mindfulness training may mitigate trajectories of persistent distress extending into the postpartum period.

A notable contribution of the present study is the demonstration of improved marital satisfaction following the mindfulness intervention. While most perinatal mindfulness research has focused primarily on individual psychological outcomes, the current findings suggest that relational functioning may also benefit. Mindfulness has been associated with enhanced empathy, emotional attunement, and constructive communication within intimate relationships [12, 13], and prior studies in non‐perinatal populations have reported positive associations between mindfulness and relationship satisfaction [28, 29, 30, 31]. This interpretation is consistent with interpersonal theories of mindfulness, which propose that increased self‐awareness and reduced emotional reactivity can improve interpersonal sensitivity and responsiveness. In the context of high‐risk pregnancy, these changes may be especially important because couples often face increased stress, uncertainty, and changes in relational roles. In the context of high‐risk pregnancy and hospitalization, where relational stress may intensify due to uncertainty and role strain, improved emotion regulation and reduced reactivity may facilitate more supportive and less conflictual partner interactions. The concurrent increase in mindfulness alongside improvements in marital satisfaction is consistent with evidence linking FFMQ‐assessed mindfulness to broader indicators of psychological well‐being and relational adjustment [27, 32].

Taken together, these findings support a dual intrapersonal–interpersonal pathway through which mindfulness may exert its effects. At the intrapersonal level, mindfulness enhances awareness and emotional regulation, thereby reducing psychological distress manifested through anxiety and depressive symptoms. At the interpersonal level, improved regulation and reduced cognitive reactivity may foster more adaptive communication patterns and relational responsiveness, contributing to greater marital satisfaction. Thus, the observed effects may be understood as resulting from both individual‐level changes in emotional regulation and relationship‐level improvements in communication and responsiveness. These complementary pathways may explain why mindfulness training was associated with improvements in both individual psychological outcomes and marital satisfaction in the present study. The relatively large effect sizes observed in this study may reflect the heightened psychological vulnerability of hospitalized high‐risk pregnant women, for whom even brief psychosocial interventions can yield meaningful improvements. Nonetheless, these effect sizes should be interpreted cautiously in light of the modest sample size and single‐center design, and replication in larger multicenter trials is warranted.

From a clinical perspective, the findings suggest that brief (20–25 min) structured MBSR sessions are feasible for integration into inpatient obstetric settings. As a non‐pharmacological and low‐cost intervention, mindfulness training may be particularly valuable for pregnant women for whom pharmacological options are limited or undesirable. Integrating mindfulness‐based approaches into routine inpatient or high‐risk prenatal care may represent a practical strategy for supporting both maternal mental health and couple functioning during a critical developmental period.

## 6 Limitations and Future Directions

Several limitations should be acknowledged. First, the study was conducted at a single center with a relatively modest sample size, which may limit generalizability. Second, the 4 weeks postpartum period was relatively short, and longer‐term outcomes beyond 4 weeks postpartum remain unknown. Third, all outcomes were assessed using self‐report measures, which may be subject to reporting bias. Additionally, marital satisfaction was assessed solely from the mothers' perspective. The absence of partner‐reported marital satisfaction data limits conclusions regarding dyadic relationship change. Future research should incorporate partner‐reported and observational measures of relational functioning, extend follow‐up durations beyond 4 weeks postpartum, and recruit larger multicenter samples to confirm and expand upon the present findings.

## 7 Conclusion

The present study provides evidence suggesting that an 8‐week MBSR program may be effective in enhancing mindfulness and marital satisfaction while reducing psychological distress, including anxiety and depressive symptoms, among hospitalized women with high‐risk pregnancies. These findings highlight the potential value of integrating structured mindfulness‐based interventions into inpatient obstetric care as part of a comprehensive psychosocial support strategy.

## Author Contributions

**Solmaz Heydarifard:** conceptualization, investigation, methodology, project administration. **Faezeh Safaralinezhad:** investigation, methodology. **Atefeh Safaralinezhad:** conceptualization, writing – original draft, investigation, methodology, validation, writing – review and editing, project administration.

## Funding

The authors have nothing to report.

## Ethics Statement

The ethics committee of Qazvin University of Medical Sciences (Approval No: IR.QUMS. REC.1403.355) approved the study.

## Conflicts of Interest

The authors declare no conflicts of interest.

## Transparency Statement

The corresponding author (AS) affirms that this manuscript is an honest, accurate, and transparent account of the study being reported; that no important aspects of the study have been omitted; and that any discrepancies from the study as planned have been explained.

## Supporting information


Supporting File

## Data Availability

The data that supports the findings of this study are available in the supplementary material of this article. All datasets used and/or analyzed during the current study are within the article.
