# Supplementary material for: Effects of a Brief Inpatient Mindfulness‐Based Stress Reduction Program on Psychological Distress and Marital Satisfaction in High‐Risk Pregnancy: A Randomized Controlled Trial
Source: Health Sci Rep. 2026 Sep 23;9(10):e73154. doi: 10.1002/hsr2.73154 (PMC13598674; doi:10.1002/hsr2.73154)
Supplement: Supplementary file 1 [file HSR2-9-e73154-s001.docx]

| **Characteristic**  Table S1. Baseline demographic and obstetric characteristics of participants included in the final analysis. | **Intervention (n=27)** | **Control (n=26)** | **p-value** |
| --- | --- | --- | --- |
| **Maternal age (years), mean ± SD** \| \| \| | 28.59 ± 5.04 | 29.85 ± 3.78 | 0.307 |
| **Gravidity, mean ± SD** \| \| \| | 2.46 ± 0.96 | 2.36 ± 0.87 | 0.693 |
| **Number of living children, mean ± SD** \| \| \| | 2.11 ± 0.79 | 2.07 ± 0.72 | 0.848 |
| **Hospitalization period, mean ± SD** \| \| \| | 5.90 ± 0.62 | 5.61 ± 0.74 | 0.129 |
| **History of abortion, n (%)** |  |  | 0.806 |
| Yes | 3(11.1) | 4(15.4) |  |
| No | 24(88.9) | 22(84.6) |  |
| **Education level, n (%)** \| \| \| |  |  | 0.646 |
| • High school or less \| \| \| | 3 (11.1) | 5 (19.20) |  |
| • Diploma \| \| \| | 7 (25.9) | 9 (34.6) |  |
| • Bachelor's degree \| \| \| | 12 (44.4) | 9 (34.6) |  |
| • Graduate degree or higher \| \| \| | 5 (18.5) | 3 (11.5) |  |
| **Employment status (participant), n (%)** |  |  | 0.832 |
| • Unemployed | 7 (25.9) | 5 (19.2) |  |
| • Housewife | 9 (33.3) | 10 (38.5) |  |
| • Employed | 11 (40.7) | 11 (42.3) |  |
| **Employment status (spouse), n (%)** |  |  | 0.338 |
| • Unemployed | 1 (3.7) | 2 (7.7) |  |
| • Employed | 24 (88.9) | 24 (92.3) |  |
| • Retired | 2 (7.4) | 0 (0.0) |  |
| **Economic status, n (%)** |  |  | 0.886 |
| • Low | 6 (22.2) | 6 (23.1) |  |
| • Moderate | 18 (66.7) | 16 (63.5) |  |
| • High | 3 (11.1) | 4 (15.4) |  |
| **Pregnancy intention, n (%)** |  |  | 0.961 |
| • Unintended | 3 (11.1) | 3 (11.5) |  |
| • Intended | 24 (88.9) | 23 (88.5) |  |

**Note:** Data are presented as mean ± SD or n (%). Percentages are column percentages. Continuous variables were compared using the independent-samples t test, and categorical variables were compared using Pearson's chi-square test. A two-sided p value < 0.05 was considered statistically significant.
